# The Role of Biomarkers for Coronary Artery Disease Detection in an Australian Rapid Access Chest Pain Assessment Clinic

**DOI:** 10.3390/jcm15020832

**Published:** 2026-01-20

**Authors:** Marwan Shawki, Neshi Weerasooriya, Anthony Salib, Hussein Al-Fiadh, Chantelle Zoumberis, Karen Sanders, Suranga Weerasooriya, Ali Al-Fiadh

**Affiliations:** 1 Department of Cardiology, Austin Health, 145 Studley Road, Heidelberg, Melbourne, VIC 3084, Australia; marwan.shawki@unimelb.edu.au (M.S.); chantelle.zoumberis@austin.org.au (C.Z.); karen.sanders@austin.org.au (K.S.); 2 Department of Medicine, University of Melbourne, Melbourne, VIC 3052, Australia; 3 School of Medicine, University of Adelaide, Adelaide, SA 5000, Australia; neshi.weerasooriya@student.adelaide.edu.au; 4 School of Medicine, Monash University, Melbourne, VIC 3168, Australia; half0001@student.monash.edu; 5 Advara HeartCare, Adelaide, SA 5062, Australia; suranga.weerasooriya@advaraheartcare.com

**Keywords:** rapid access chest pain clinic, coronary artery disease, CT coronary angiography, high-sensitivity C-reactive protein, low-density lipoprotein cholesterol

## Abstract

**Background/Objectives:** The Rapid Access Chest Pain Assessment Clinic (RACPAC) streamlines the evaluation of low-to-intermediate risk chest pain and helps avoid unnecessary hospitalisation. Biomarkers {low-density lipoprotein cholesterol (LDL-c) and high-sensitivity C-reactive protein (hsCRP)} are established cardiovascular risk markers. Yet, their diagnostic value for stable coronary artery disease (CAD) in RACPAC remains uncertain. Therefore, we aimed to determine the utility of biomarkers in predicting the presence of CAD in the RACPAC setting. **Methods:** A retrospective cohort study of consecutive adults attending RACPAC between 2012 and 2021. Multivariable logistic regression and receiver operating characteristic analyses, including prespecified subgroup and sensitivity analyses, were used to evaluate the predictive value of hsCRP and LDL-c for the presence of CAD detected on CT Coronary Angiogram (CTCA) or Treadmill Stress Echocardiography (TSE) as the primary outcome. **Results:** 3569 patients were included in this study, the mean age was 55.4 ± 11.3 years, and 48.8% were female; 37.4% had hypertension, while 39.5% had dyslipidemia. The mean LDL-c was 3.1 ± 0.9 mmol/L, and the median hsCRP was 1.9 mg/L (IQR 0.9 to 3.8). The regression analysis for the primary outcome showed that neither hsCRP nor LDL-c predicted CAD on CTCA (hsCRP OR 1.00, 95% CI 0.99 to 1.02, *p* = 0.70; LDL-c OR 1.16, 95% CI 0.97 to 1.39, *p* = 0.11). On TSE, hsCRP was not associated with CAD, while LDL-c showed an inverse association with CAD (hsCRP OR 0.98, 95% CI 0.83 to 1.00, *p* = 0.78; LDL-c OR 0.44, 95% CI 0.21 to 0.87, *p* = 0.02). ROC analysis showed AUC 0.553 for log hsCRP (95% CI 0.501 to 0.606) and 0.508 for LDL-c (95% CI 0.450 to 0.566), with *p* = 0.2756. **Conclusions:** In a large real-world RACPAC cohort, neither elevated hsCRP nor LDL-c predicted the presence of coronary artery disease in the rapid access chest pain clinic (RACPAC) cohort. In contrast, CT coronary angiography (CTCA) demonstrated superior diagnostic accuracy compared with treadmill stress echocardiography (TSE) in this setting.

## 1. Introduction

The Rapid Access Chest Pain Assessment Clinic (RACPAC) was introduced as a system-level solution to rapidly evaluate suspected angina in the outpatient setting, reducing hospital admissions while expediting investigation and treatment. The model has since been adopted internationally, with evidence of reduced emergency department crowding, lower costs, and high patient satisfaction [1,2,3,4]. In Australia, several large tertiary centres have established RACPACs, but systematic data remain limited [4]. Despite the proven safety and effectiveness of this model, the increasing number of chest pain presentations and their economic burden highlight the need for more refined triage strategies to reduce waiting times and minimise unnecessary cardiac investigations where appropriate [5]. The biomarker revolution in cardiovascular prevention has transformed the understanding of coronary artery disease (CAD) risk. Low-density lipoprotein cholesterol (LDL-c) is firmly established as a causal driver of atherosclerosis, supported by numerous prospective cohort data and lipid-lowering trials [6,7,8]. Current European Society of Cardiology (ESC) and European Atherosclerosis Society (EAS) dyslipidaemia guidelines therefore recommend intensive LDL-c lowering proportional to absolute cardiovascular risk [9]. High-sensitivity C-reactive protein (hsCRP), a marker of systemic inflammation, is also strongly associated with coronary risk [10,11,12,13]. In the Women’s Health Study, hsCRP independently predicted myocardial infarction and stroke, including in individuals with normal LDL-c [14]. The JUPITER trial showed that rosuvastatin reduced vascular events in people with elevated hsCRP despite normocholesterolaemia [11], and CANTOS provided proof of concept that targeting interleukin 1β reduced recurrent events independently of LDL-c [12]. These data raise an important question: can LDL-c or hsCRP be used not only for risk prediction, but also for diagnostic discrimination in symptomatic patients with stable chest pain, particularly in the RACPAC setting where efficient triage is critical? At the same time, advances in cardiac imaging have reshaped the diagnostic pathway for suspected CAD. Functional stress testing, such as treadmill stress echocardiography (TSE) and myocardial perfusion scintigraphy (MPS), was historically the mainstay. However, trials such as PROMISE and SCOT HEART demonstrated advantages of anatomic imaging with CT coronary angiography (CTCA) in diagnostic certainty and downstream management [15,16]. CTCA identifies both obstructive and non-obstructive disease, characterises plaque morphology, and detects extracardiac pathology. The 2019 ESC chronic coronary syndrome guidelines now recommend CTCA as the preferred first-line test for most patients with suspected CAD, reserving functional testing for those in whom CTCA is contraindicated or unavailable [17]. Against this background, we aimed to evaluate whether hsCRP or LDL-c can provide a diagnostic value for the presence of CAD in symptomatic, low-to-intermediate-risk patients attending an Australian RACPAC, and to examine how these biomarkers perform alongside contemporary imaging modalities (CTCA, TSE) in this setting.

## 2. Methods

### 2.1. Study Design and Setting

We conducted a retrospective observational cohort study of consecutive patients referred to a tertiary hospital’s RACPAC in Melbourne, Australia, between July 2012 and July 2021. The clinic provides rapid outpatient evaluation for patients with new or recurrent chest pain considered low-to-intermediate risk after initial assessment in primary care or the emergency department. This model parallels UK and European rapid access chest pain services while incorporating Australian referral pathways.

### 2.2. Inclusion and Exclusion Criteria

Adults aged 18 years or older with chest pain of suspected cardiac origin were included if they were classified as low-to-intermediate risk after initial evaluation, defined by normal troponin, absence of acute ST segment changes, and haemodynamic stability, and were referred by the emergency department, following inpatient discharge, or by a general practitioner. Patients were excluded if they had known coronary artery disease, including prior myocardial infarction, percutaneous coronary intervention, or coronary artery bypass grafting, were under ongoing specialist cardiology follow-up, or had incomplete data for both biomarkers and imaging outcomes.

### 2.3. Data Collection

Baseline data were extracted from clinical records and included demographics, cardiovascular risk factors, Family history of CAD, statin use, and modality of cardiac imaging requested at RACPAC. LDL-c was measured in mmol/L using standard enzymatic assays, and hsCRP was measured in mg/L using an immunoassay. Both biomarkers were obtained at the initial RACPAC visit. Cardiac imaging results included outcomes of TSE, CTCA, MPS, and invasive coronary angiography (ICA). Data were extracted from the electronic medical record by three co-authors (MS, HA, AS) using a standardised data collection template. To ensure data quality, 5% of records were randomly selected for independent audit by (AA) and cross-checked against the source files to verify the accuracy of data extraction.

### 2.4. Ethics Approval

The Austin Health Ethics Committee approved the study (HREC/92453/Austin-2023).

### 2.5. Imaging Modalities and Biomarkers

TSE was performed using the Bruce protocol, where feasible, and terminated according to standard endpoints. A positive test was defined as an inducible wall motion abnormality, and submaximal studies were classified as inconclusive. CTCA was performed on 64 or 128-slice multidetector scanners using prospective or retrospective ECG gating. Reports used the Coronary Artery Disease Reporting and Data System (CAD RADS 2.0) with grades from 0 to 5 and coronary artery calcium scoring. For descriptive analyses, coronary artery disease was defined as CAD RADS 3 or higher, whereas for logistic regression analyses evaluating obstructive disease, it was defined as CAD RADS 4 or higher. MPS was performed with pharmacological stress in patients unable to exercise, and a positive study was defined as a reversible perfusion defect. On ICA, obstructive CAD was defined as at least 50% diameter stenosis in a major epicardial vessel. Elevated LDL-c was defined as 2.0 mmol/L or higher, and elevated hsCRP as 1.1 mg/L or higher.

### 2.6. Outcomes Measured

The primary outcome was the predictive value of elevated hsCRP or LDL-c in identifying the presence of CAD on CTCA or TSE. Secondary outcomes were the pattern of cardiac imaging utilisation, the proportion of patients progressing from non-invasive imaging to invasive coronary angiography, the rate of coronary revascularisation with percutaneous coronary intervention (PCI) or coronary artery bypass grafting (CABG), and the rate of re-presentation to the RACPAC within 12 months following index discharge from the clinic.

### 2.7. Statistical Analysis

Continuous variables were summarised as means and standard deviations or medians and interquartile ranges, as appropriate. Categorical variables were presented as numbers and percentages. Between-group comparisons were performed using Student’s *t*-test, the Mann–Whitney U test, or the chi-square test, as appropriate. Multivariable logistic regression was used to assess the independent associations of elevated LDL-c or hsCRP with obstructive CAD on CTCA (defined as CADRADS 4 or higher), regional wall motion abnormalities on TSE. Controlling for age, sex, hypertension, diabetes, smoking status, and statin use at the time of initial RACPAC assessment. Prespecified subgroup analyses were conducted by sex, age group (less than 45 years versus 45 years or older), and statin status. A sensitivity analysis excluding patients with CRP above 10 mg/L was performed to reduce potential confounding from acute inflammation. Receiver operating characteristic (ROC) analysis was performed to assess the ability of hsCRP and LDL-c to discriminate the presence of CAD detected on CTCA or TSE. Areas under the curve were compared, and optimal cutoffs for each biomarker were determined using the Youden Index. All analyses were performed using Stata version 17 (StataCorp LLC, College Station, TX, USA), and statistical significance was set at *p* < 0.05.

## 3. Results

### 3.1. Cohort

A total of 3569 patients were included. The mean age was 55.4 ± 11.3 years, and 48.8% were female. Hypertension was present in 37.4%, dyslipidaemia in 39.5%, diabetes in 14.5%, and 39.7% had an ex or current smoking status. Mean LDL-c was 3.1 ± 0.9 mmol/L, and median hsCRP was 1.9 mg/L (IQR 0.9 to 3.8) (Table 1).

### 3.2. Cardiac Imaging Utilisation and Outcomes

Among patients undergoing non-invasive testing, a subset proceeded to invasive coronary angiography. As some patients required more than one imaging modality to reach a diagnosis, the total number of investigations exceeded the number of individuals assessed. Following TSE, 67 patients (4.0%) underwent ICA, of whom 55 (82%) had CAD confirmed. After CTCA, 150 patients (9.5%) proceeded to ICA, with CAD confirmed in 146 (97%). After MPS, 18 patients (10.1%) underwent ICA, with CAD confirmed in 16 (approximately 89%). Among those referred directly to ICA, CAD was present in 230 of 284 procedures (81%). These positive predictive values for TSE, CTCA, and MPS are summarised in Table 2.

### 3.3. Biomarkers and Outcomes

**Figure 1:**
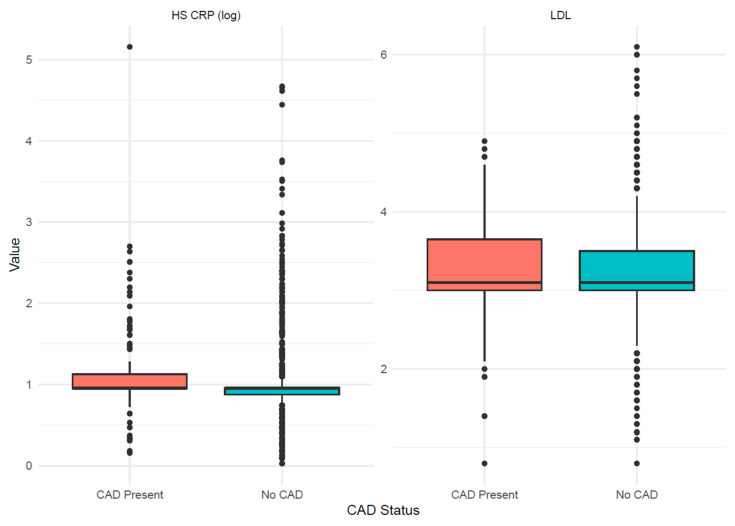
Distribution of biomarker by coronary artery disease status. Boxplots compare log-transformed HsCRP and LDL-c values between patients with coronary artery disease present and those with no coronary artery disease. Coronary artery disease status is shown on the *x*-axis, and biomarker values are shown on the *y*-axis. The central line represents the median, the box represents the interquartile range, whiskers represent the spread of values, and individual points represent observed values, including outliers. HsCRP indicates high-sensitivity C-reactive protein, and LDL-c indicates low-density lipoprotein cholesterol.

**Figure 2:**
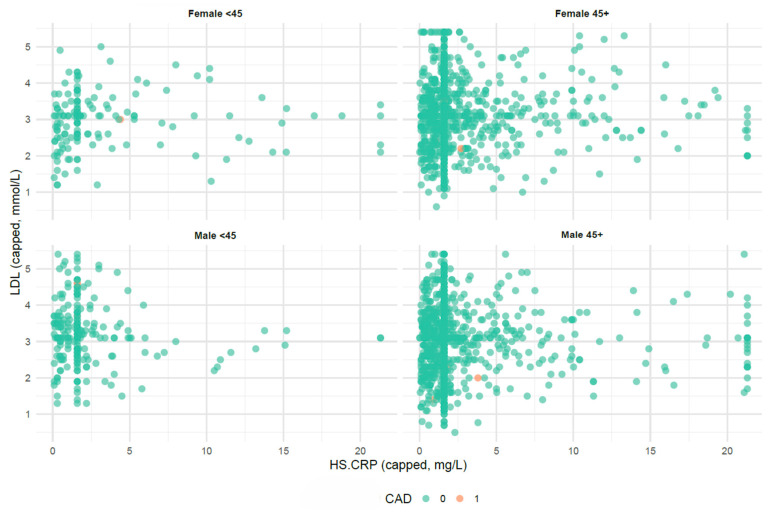
Subgroup analysis of biomarkers by age and sex. Scatterplots show HsCRP values on the x-axis and LDL-c values on the y-axis, stratified by sex and age group, younger than 45 years and 45 years or older. Each point represents an individual patient, with colour indicating CAD status. hsCRP indicates high-sensitivity C-reactive protein, LDLc low-density lipoprotein cholesterol, and CAD coronary artery disease.

In multivariable logistic regression models adjusted for age, sex, hypertension, diabetes, smoking status, and statin use, hsCRP was associated with an odds ratio (OR) of 1.00 (95% CI 0.99 to 1.02, *p* = 0.70) and 0.98 (95% CI 0.83 to 1.00, *p* = 0.78) for the presence of CAD on CTCA and TSE, respectively. LDL-c was associated with an OR of 1.16 (95% CI 0.97 to 1.39, *p* = 0.11) and 0.44 (95% CI 0.21 to 0.87, *p* = 0.02) for the presence of CAD on CTCA and TSE, respectively. For revascularisation and re presentation within 12 months, the ORs were 1.00 (95% CI 0.95 to 1.00, *p* = 0.81) and 1.00 (95% CI 0.99 to 1.00, *p* = 0.90) for hsCRP, and 0.95 (95% CI 0.64 to 1.38, *p* = 0.81) and 1.20 (95% CI 0.78 to 1.77, *p* = 0.39) for LDL-c (Table 3, Figure 1). Subgroup analyses by sex and age (<45 vs. ≥45 years) showed neither hsCRP nor LDL-c was associated with the presence of CAD on CTCA or TSE, consistent with the main results of the regression model (Table 3, Figure 2). Among statin-treated patients, ORs for evidence of CAD on CTCA were 0.99 (95% CI 0.92–1.03; *p* = 0.57) for hsCRP and 1.07 (95% CI 0.76–1.50; *p* = 0.70) for LDL-c. Among patients not taking statins, the OR for evidence of CAD on TSE was 0.46 (95% CI 0.21–0.97; *p* = 0.05) for LDL-c. After excluding patients with hsCRP > 10 mg/L, hsCRP showed OR 0.99 (95% CI 0.91–1.07; *p* = 0.38) for evidence of CAD on CTCA and OR 0.98 (95% CI 0.87–1.13; *p* = 0.13) for evidence of CAD on TSE.

### 3.4. Receiver Operating Characteristic Analysis

**Figure 3:**
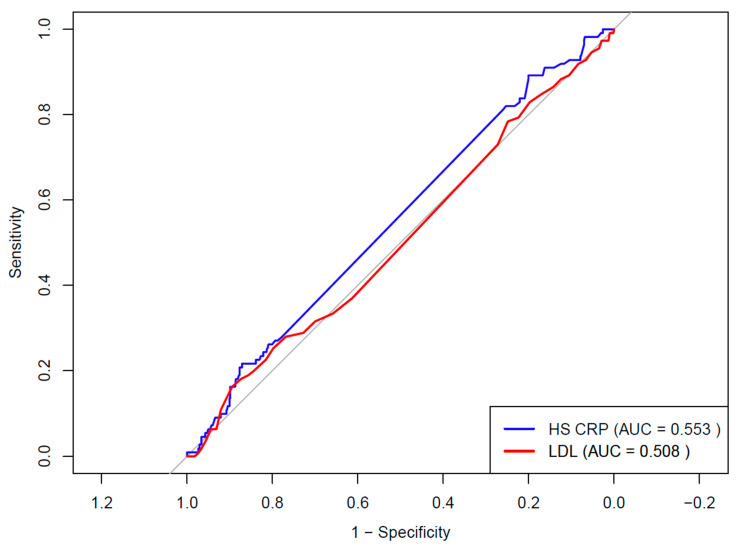
Receiver operating characteristic curves for biomarkers for coronary artery disease detection. Receiver operating characteristic curves showing the discriminative performance of HsCRP and LDL-c for the presence of coronary artery disease. The blue line represents HsCRP with an AUC of 0.553, and the red line represents LDL-c with an AUC of 0.508, with the diagonal line indicating no discrimination. AUC indicates area under the curve, hsCRP high-sensitivity C-reactive protein, and LDL-C low-density lipoprotein cholesterol.

Receiver Operating Characteristic analysis showed an Area Under the Curve (AUC) of 0.553 (95% CI 0.501 to 0.606) for log-transformed hsCRP and 0.508 (95% CI 0.450 to 0.566) for LDL-c, with no significant difference between curves (*p* = 0.2756) (Figure 3). The Youden Index identified an optimal cutoff of 4.15 for hsCRP mg/L (sensitivity 71%, specificity 16.2%).

## 4. Discussion

Our study provides one of the largest real-world evaluations of hsCRP and LDL-c in low-to-intermediate-risk patients attending a RACPAC in Australia, examined alongside contemporary imaging pathways and downstream management outcomes. Our main findings were that neither hsCRP nor LDL-c provided independent predictive value for the presence of CAD detected on CTCA or TSE. In contrast, CTCA had the highest CAD detection rate and the greatest predictive value for subsequent revascularisation among the non-invasive imaging modalities.

LDL-c is a well-established causal risk factor for atherosclerotic cardiovascular disease [6,7,8,9]. Yet, in our cohort, a single cross-sectional LDL-c value had limited ability to discriminate established CAD in low-to-intermediate-risk symptomatic outpatients. Several factors may explain this apparent discrepancy. First, RACPAC referrals are preselected as low-to-intermediate risk, which likely narrows baseline LDL-c variability and introduces selection bias. Second, more than one-quarter of the cohort were already receiving statin therapy at referral, which may have attenuated LDL-c levels in patients with established disease. However, our subgroup analyses did not demonstrate a clear modifying effect. Third, a single LDL-c measurement does not capture long-term lipid burden, whereas atherosclerosis develops over decades. Fourth, Mendelian randomisation studies support a dose-dependent relationship between lifelong LDL-c exposure and CAD risk [8]. Finally, atherogenesis is multifactorial, with contributions from endothelial dysfunction, inflammation, lipoprotein(a), apolipoprotein B burden, and genetic susceptibility, which may allow CAD to occur even in the setting of low to normal LDL-c [18,19]. In a large cohort of the Danish Heart registry that looked into symptomatic patients who underwent CTCA, it was also demonstrated that single-time-point LDL-c may not track well with the presence or absence of calcified or non-calcified plaque, reinforcing the limitation of cross-sectional LDL-c for disease detection [20]. Taken together, LDL-c remains central to risk assessment and preventive therapy but appears to have limited diagnostic utility for established CAD in the RACPAC setting.

High-sensitivity C-reactive protein reflects systemic inflammatory activity and has demonstrated prognostic value for CAD in large cohorts [11,12,13,14]. In contrast, in our symptomatic low-to-intermediate risk clinic population, hsCRP showed limited value in detecting stable CAD. Our findings are in line with a large Swedish cross-sectional study of men and women aged 50 to 64 years, in which elevated hsCRP was a poor predictor of the presence of CAD detected via CTCA, after adjusting for traditional cardiovascular risk factors [21]. This likely reflects an important distinction between risk prediction and disease detection. hsCRP is better suited to identifying individuals at higher risk of future plaque instability-related events, whereas imaging is required to define current plaque burden and obstructive disease. Our findings are consistent with prior smaller studies in emergency chest pain cohorts, in which hsCRP showed insufficient sensitivity and specificity for obstructive CAD [17,22].

The inverse association between LDL-c and a positive TSE should be interpreted cautiously. TSE reflects functional ischemia, whereas LDL-c reflects risk exposure, and the subgroup undergoing TSE was clinician-selected. Differential referral to CTCA, confounding by indication, and lipid-lowering among higher-risk patients may produce an apparent inverse association that does not imply a protective effect of higher LDL-c.

In our study, CTCA emerged as the most effective diagnostic tool, identifying coronary artery disease in approximately one-third of tested patients. This aligns with major trial data demonstrating the diagnostic and management advantages of anatomic imaging. PROMISE and SCOT HEART showed that CTCA improves diagnostic certainty, guides therapy, and is associated with favourable clinical outcomes [15,16]. The ISCHEMIA trial, although focused on invasive versus conservative management strategies, further emphasised the importance of defining coronary anatomy to inform therapeutic decisions [19]. The strength of CTCA lies in its ability to detect both obstructive and non-obstructive disease, identify early atherosclerosis that may be missed by functional testing, and provide plaque characterisation, including high-risk features such as positive remodelling and low attenuation, which have been linked to adverse events [17,23,24]. By comparison, treadmill stress echocardiography in our cohort showed lower test positivity and lower conversion to invasive angiography, which may reflect reduced sensitivity for non-obstructive or multivessel disease [25]. In addition, emerging evidence shows that, in patients with stable chest pain, CTCA is more cost-effective than TSE for improving health-related quality of life [26] and may provide added value by detecting early coronary plaque, enabling timely initiation of preventive therapies such as statins and reinforcing lifestyle modification to reduce future cardiovascular risk. Overall, this imaging pattern aligns with ESC guidance that supports CTCA as a first-line investigation for many patients with suspected CAD in the chronic coronary syndrome pathway [17].

Our findings highlight two related but distinct paradigms in chest pain assessment. Biomarkers such as LDL-c and hsCRP are valuable for estimating future cardiovascular risk. Still, they do not reliably identify current coronary disease burden in a symptomatic, low-to-intermediate risk RACPAC population. In contrast, imaging defines the current anatomical and functional state of disease and directly informs immediate management decisions. In the RACPAC context, where the priority is efficient triage of chest pain, disease detection is more clinically actionable than risk prediction. This does not diminish the importance of biomarkers for long-term prevention. An integrated approach that combines imaging-defined disease with biomarker-guided preventive therapy may provide the most coherent pathway for ongoing care.

### 4.1. Clinical Implications

These data suggest that LDL-c and hsCRP should not be used for diagnostic triage in RACPAC. Their principal role remains in preventive cardiology, including risk stratification, treatment selection, and longer-term prognostication, rather than in acute or subacute chest pain evaluation. CTCA should be prioritised as the first-line imaging test where available and appropriate, in keeping with contemporary guideline-based pathways, with treadmill stress echocardiography reserved for patients in whom CTCA is contraindicated or not feasible. Additionally, CTCA routine hsCRP testing may be avoided in this clinic setting to reduce unnecessary cost. In contrast, lipid testing should be retained to support downstream preventive management and optimisation of lipid-lowering therapy.

### 4.2. Limitations

This study has several limitations. Its retrospective observational design limits causal inference and is vulnerable to unmeasured or residual confounding. Residual confounding may persist due to incomplete capture of medication adherence and the intensity and duration of lipid-lowering therapy, including recent treatment changes and concomitant lipid-lowering agents. Although we adjusted for statin use at baseline, granular data on dose intensity, duration, and adherence were not reliably available, and other unmeasured factors such as intercurrent infection, inflammatory comorbidities, and use of anti-inflammatory medications may have influenced both hsCRP and LDL-c values. In addition, not all patients underwent both CTCA and TSE, and test selection was clinician-directed rather than protocolized, introducing potential verification and workup bias. This may have resulted in systematic differences in baseline risk between those referred for CTCA versus TSE, which should be considered when interpreting comparative test performance and associations with imaging outcomes.

As a single-centre RACPAC cohort enriched for low-to-intermediate-risk referrals, selection and referral bias are likely, and findings may not generalise to higher-risk populations or centres with different triage thresholds and imaging availability. Biomarkers were measured at a single time point, which does not capture cumulative exposure or longitudinal change, particularly in patients already treated with statins. Imaging results were obtained from clinical reports rather than from central adjudication, and changes in scanners, protocols, and reporting standards from 2012 to 2021 may have introduced heterogeneity and misclassification. Finally, outcomes focused on imaging findings and short-term outcomes such as revascularisation and re-presentation, with limited assessment of longer-term hard endpoints such as myocardial infarction and cardiovascular death.

### 4.3. Future Direction

Future studies should prospectively validate these findings in multicentre RACPAC cohorts and link clinic data to longer-term outcomes such as myocardial infarction and mortality. Further work could test whether simple anthropometric measures, including waist circumference and waist-to-hip ratio, and thoracic morphology measures, such as anteroposterior thoracic diameter or modified Haller index, add incremental value to pretest risk assessment and imaging-based triage. These measures may help identify low-risk phenotypes (including mitral valve prolapse patterns) and reduce false-positive functional testing [27,28]. Further work could also explore novel diagnostic biomarker panels using multi-omics approaches and assess whether combining CT coronary angiography plaque characteristics with lipid or inflammatory markers improves risk stratification. Artificial intelligence-enabled CT coronary angiography quantification of plaque burden may also refine diagnostic and prognostic pathways.

## 5. Conclusions

In a large real-world RACPAC cohort, neither LDL-c nor hsCRP predicted CAD detected by CT coronary angiography or treadmill stress echocardiography. Cardiac imaging, particularly CT coronary angiography, remains the cornerstone for diagnostic triage of stable chest pain, while these biomarkers are better suited to longer-term risk stratification and therapeutic guidance.

## Figures and Tables

**Table 1:** Baseline characteristics.

Variable	Overall (n = 3569)	Male (n = 1825)	Female (n = 1744)
Age, years, mean ± SD	55.4 ± 11.3	56.7 (10.9)	54.0 ± 11.5
Hypertension, %	37.4%	38.9%	36.0%
Dyslipidemia, %	39.5%	41.7%	37.2%
Diabetes %	14.5%	16.2%	12.6%
Smoking, %	39.7%	44.3%	34.8%
Family History CAD, %	48.4%	47.8%	49.0%
LDL-c (mmol/L), mean ± SD	3.1 ± 0.9	3.2 ± 0.9	3.0 ± 0.9
Statin use, %	27.5%	28.8%	26.1%
HSCRP, mg/L, median (IQR)	1.9 (0.9–3.8)	1.7 (0.8–3.2)	2.2 (1.0–4.1)

Data are presented as mean (SD) for age and LDL-c, median (IQR) for hsCRP, and percentage for categorical variables. CAD indicates coronary artery disease, LDL-c low-density lipoprotein cholesterol, and hsCRP high-sensitivity C-reactive protein.

**Table 2:** Cardiac imaging utilisation and outcomes.

Modality	N (%)	Positive Test n (%)	Conversion to ICA n (%)	CAD Confirmed on ICA n (%)	PCI/CABG n (%)
TSE	1687 (45.2)	50 (3)	67 (4)	55/67 (82)	450 (26.7)
CTCA	1579 (42.3)	584 (37)	150 (9.5)	146/150 (87)	546 (34.6)
MPS	179 (4.8)	28 (16)	18 (10.1)	16/18 (89)	36 (20)
ICA	284 (7.6)	230 (81)	-	230/284 (81)	95 (33.3)

Cardiac imaging utilisation and downstream procedures. Data are presented as numbers (percentages). Conversion to ICA refers to the proportion of patients undergoing invasive coronary angiography following the index non-invasive modality. PCI or CABG refers to the proportion of patients undergoing revascularisation. TSE indicates treadmill stress echocardiography, CTCA CT coronary angiography, MPS myocardial perfusion scintigraphy, ICA invasive coronary angiography, PCI percutaneous coronary intervention, and CABG coronary artery bypass grafting.

**Table 3:** Multivariable models: biomarkers and outcomes.

Outcome	hsCRP OR (95% CI)	*p*-Value	LDL-C OR (95% CI)	*p*-Value
CAD on CTCA	1.00 (0.99–1.02)	0.70	1.16 (0.97–1.39	0.11
CAD on TSE	0.98 (0.83–1.00)	0.78	0.44 (0.21–0.87)	0.02
Revascularisation	1.00 (0.95–1.00)	0.81	0.95 (0.64–1.38)	0.81
Re-presentation	1.00 (0.99–1.00)	0.90	1.20 (0.78–1.77)	0.39
CAD in Male	1.00 (0.99–1.03)	0.62	1.10 (0.84–1.44)	0.49
CAD in Female	1.01 (0.97–1.05)	0.54	1.25 (0.97–1.62)	0.09

Multivariable models of biomarkers and clinical outcomes. Data are presented as odds ratios with a 95% confidence interval. Models evaluate associations of hsCRP and LDL-c with CAD on CTCA, inducible ischemia on TSE, revascularisation, and re-presentation, adjusted for age, sex, hypertension, diabetes, smoking status, and statin use. hsCRP indicates high-sensitivity C-reactive protein, LDL-c low-density lipoprotein cholesterol, CAD coronary artery disease, CTCA CT coronary angiography, TSE treadmill stress echocardiography, OR odds ratio, and CI confidence interval.

## Data Availability

De-identified data that support the findings of this study are available from the corresponding author upon reasonable request and subject to institutional ethics approval.

## Abbreviations

RACPAC	Rapid Access Chest Pain Assessment Clinic
LDL-c	Low-density lipoprotein cholesterol
hsCRP	High-sensitivity C-reactive protein
CAD	Coronary artery disease
CTCA	CT coronary angiography
TSE	Treadmill stress echocardiography
ROC	Receiver operating characteristic
AUC	Area under the curve
ESC	European Society of Cardiology
EAS	European Atherosclerosis Society
MPS	Myocardial perfusion scintigraphy
ECG	Electrocardiogram
CAD RADS	Coronary Artery Disease Reporting and Data System
ICA	Invasive coronary angiography
PCI	Percutaneous coronary intervention
CABG	Coronary artery bypass grafting
